# Effects of liraglutide on hemodynamic parameters in patients with heart failure

**DOI:** 10.18632/oncotarget.18570

**Published:** 2017-06-19

**Authors:** Jin Ying Zhang, Xin Yun Wang, Xiang Wang

**Affiliations:** ^1^ Department of Emergency, Binzhou Medical University Hospital, Binzhou, Shandong, China

**Keywords:** glucagon-like peptide-1, heart failure, hemodynamic, pulse indicator continuous cardiac output, left ventricular function

## Abstract

Glucagon-like peptide-1 analogues improve left ventricular function in patients with acute myocardial infarction. This study aimed to evaluate the effects of liraglutide on hemodynamic parameters in patients with heart failure. A total of 78 patients with heart failure were enrolled in this study between August 2014 and November 2015. Of these, 52 patients were randomized 1:1 to receive either liraglutide or placebo for 7 days. Hemodynamic measurements were made using transpulmonary thermodilution and arterial pulse contour analysis. At 7 days, the difference in change of the primary endpoint of cardiac output between the liraglutide group and control group was +1.1 1/min (95% CI +0.1 to +2.2; *P* < 0.001). Stroke volume was significantly higher in the liraglutide group compared with the control group (difference: +14.6 ml; *P* < 0.001). The difference in an increase in the left ventricular contractile index after 7 days of treatment was +210.7 mmHg/s (liraglutide versus control, 95% CI−92.1 to +501.5; *P* < 0.001). Liraglutide causes favorable changes in markers of inflammation and oxidative stress. Glucagon-like peptide-1 may be associated with improvement in left ventricular function in patients with heart failure. These findings need to be confirmed by larger invasive trials.

## INTRODUCTION

Heart failure (HF) is a major cause of morbidity and mortality worldwide [1]. Glucagon-like peptide-1 (GLP-1) is an incretin hormone that reduces plasma glucose, and has direct effects on the cardiovascular system [2]. Several studies have investigated short-term and longer-term effects of GLP-1 in HF [3, 4]. Recently, two large trials evaluated the effect of liraglutide in HF. Neither LIVE nor FIGHT could demonstrate a positive effect of liraglutide in HF [5, 6]. In both of these studies, more serious adverse cardiovascular events were observed in the liraglutide group compared to the control group. Pulse indicator continuous cardiac output (PiCCO) technology is a combination of transpulmonary thermodilution and pulse contour analysis. This technology measures hemodynamic variables (cardiac output, left ventricular ejection fraction and volume) in a fast and feasible way. This study aimed to evaluate the effects of liraglutide on hemodynamic variables in patients with HF using the PiCCO system.

## MATERIALS AND METHODS

### Study site and ethics

This was a single-center, prospective, interventional study that was conducted at the Binzhou Medical University Hospital, China. The study was approved by the Binzhou Ethics Association and the Ethics Committee of the Binzhou Medical University Hospital, and complied with the Helsinki Declaration. All of the subjects provided written informed consent to participate in the study. The trial was registered at ClinicalTrials.gov (registration number: NCT02490176).

### Study population

The study was conducted between August 2014 and November 2015. Patients with HF were eligible for inclusion in the study. Diagnosis of HF was based on an impaired ejection fraction (< 50%) [7]. Patients were also excluded for the following reasons: unconscious at presentation, valvular heart disease, cardiogenic shock, hypoglycaemia, diabetic ketoacidosis, and renal insufficiency.

### Definitions

Hypertension was defined as blood pressure ≥ 140/90 mmHg or a history of antihypertensive drug use. Diabetes was defined as a fasting blood glucose level ≥ 7 mmol/L or a history of using oral hypoglycemic drugs or insulin. Hypoglycemia was defined as plasma glucose level ≤ 3.9 mmol/L [8]. The estimated glomerular filtration rate was calculated using the Chronic Kidney Disease Epidemiology Collaboration formula [9] and renal insufficiency was defined as an estimated glomerular filtration rate of < 60 mL/min/1.73 m^2^ [10].

### Experimental treatment protocol

All patients were informed of potential risks (hypoglycemia, pancreatitis, nausea) [11] associated with GLP-1 analogues and were then required to submit written informed consent prior to being included in the study. Patients were treated with stabile doses of β-blockers, angiotensin-converting enzyme inhibitors, angiotensin-II receptor agonists, and diuretics at least 3 months prior to randomization. Patients were randomized using a computer-generated sequence to either placebo or liraglutide at a 1:1 ratio. Investigators, participants, and other study personnel were blinded to the assigned treatment for the duration of the study. Patients in the liraglutide group were treated with subcutaneous liraglutide (Novo Nordisk, Bagsvaerd, Denmark), while patients in the control group were provided subcutaneous placebo (Novo Nordisk). After admission, the patients were treated with 0.6 mg liraglutide once daily for 2 days, 1.2 mg liraglutide for another 2 days, and then 1.8 mg liraglutide for 3 days. Such dosages corresponded to 1.6, 3.2, and 4.8 pmol/kg/min, respectively, as referred for an ideal 70 kg patient. Each patient received seven injections of liraglutide or placebo. Immediately after admission, the patients received the first injection of test drug. An oral glucose tolerance test was performed before discharge (usually on day 6). Because the patients were treated with an off-label drug, Good Clinical Practice training was required for all personnel who were involved in the trial.

### Study outcomes

The primary efficacy endpoint was the effect of liraglutide on cardiac output (CO) at 7 days compared with placebo. Secondary efficacy endpoints included the effects of liraglutide on mean arterial pressure (MAP), the cardiac index (CI), stroke volume (SV), global end diastolic volume index (GEDVI), the left ventricular contractile index (dPmx), central venous pressure, serum high-sensitivity C-reactive protein (hsCRP) levels, malondialdehyde levels, and nitric oxide (NO) levels.

### Laboratory tests

Blood samples were collected and analyzed in the Department of Clinical Biochemistry according to the department’s clinical standards. The laboratory data were obtained at admission and at 7 days. Blood samples were analyzed within 2 h after collection. N-terminal pro-B-type natriuretic peptide levels were determined by enzyme immunoassay (Shionoria, Osaka, Japan). The intra-assay coefficient of variation [CV] was 1.3% and the inter-assay CV was 2.3% (normal range, < 150 pg/mL). Levels of hsCRP were measured using a sandwich enzyme-linked immunosorbent assay (ELISA) (R&D Systems Inc., Minneapolis, MN, USA). The intra-assay CV was 3.2% and the inter-assay CV was 3.1% (normal range, < 0.8 mg/dL). Serum interleukin-6 concentrations were measured using an ELISA (R&D Systems Inc., Minneapolis, MN, USA). The intra-assay CV was 2.5% and the inter-assay CV was 4.3%, (normal range, < 8 pg/mL). Superoxide dismutase (SOD) activity was estimated as inhibition of a colorimetric reaction using an assay kit (Cayman Chemicals, Ann Arbor, MI, USA). The intra-assay CV was 4.3% and the inter-assay CV was 5.1% (normal range, 129–216 U/mL). Serum malondialdehyde levels were measured using a thiobarbituric acid-reactive substance method [12] The pink adduct formed by samples was extracted in *n*-butanol. Each sample was placed in a 96-well plate and read at 535 nm in a microplate spectrophotometer reader (Benchmark Plus, Bio-Rad, Hercules, CA, USA). The intra-assay CV was 2.2% and the inter-assay CV was 4.1% (normal range, 3.46–4.66 nmol/mL). Plasma NO levels were estimated by measuring the levels of nitrate and nitrite as markers of NO bioavailability using a nitrate and nitrite colorimetric assay (Cayman Chemicals). The intra-assay CV was 2.4% and the inter-assay CV was 3.1% (normal range, 28–50 μmol/L). Nitric oxide synthase (NOS) activity was determined using a NOS colorimetric assay (Cayman Chemicals). The intra-assay CV was 2.5% and the inter-assay CV was 3.5% (normal range, 4.93–5.35 U/mL).

### PiCCO

All patients were equipped with a central venous catheter (jugular or subclavian) and a 4-F thermistor-tipped arterial catheter (PV2015L13; Pulsion Medical Systems, Munich, Germany), which was inserted into the left femoral artery and advanced to the abdominal aorta. CO, the CI, and the GEDVI were determined discontinuously by thermodilution using a triplicate injection of 15 ml of ice-cold saline that was administered through the central venous catheter. This method has also been described in detail elsewhere [13] A continuous CO catheter was inserted for 7 days and this procedure was clearly explained to the patients. All of the patients were at rest, in the decubitus position all of the time. All measurements by PiCCO were undertaken by two investigators who were both blinded to randomization.

### Reproducibility

To determine the reproducibility of the hemodynamic parameters, 20 randomly selected patients were analyzed by two independent blinded observers. The correlation coefficients of interobserver variability for CO, the CI, and SV were 0.92, 0.90, and 0.94, respectively. The correlation coefficients of intraobserver variability for CO, the CI, and SV were 0.91, 0.88, and 0.93, respectively.

### Clinical follow-up

There was a pre-specified outpatient follow-up visit per protocol at 3 months. Follow-up information on the study population was obtained during a visit by patients to the outpatient clinic or from a review of the medical records. Follow-up was completed for all patients (follow-up rate of 100%). Major adverse cardiovascular events (MACE) were defined as non-fatal myocardial infarction, hospitalization for heart failure, and cardiac death.

### Statistical analysis

In a pilot study, a difference in CO was detected between the liraglutide and control groups at 7 days (1.2 ± 1.3 l/min, *n =* 22). We assumed an average difference in CO between the liraglutide and placebo groups of 1.2 l/min (SD = 1.3). We estimated a sample size of 44 subjects in each study group to achieve a power of 90% to demonstrate superiority of the liraglutide arm over the control arm in the primary endpoint with two-tailed α < 0.05. On the assumption that approximately 90% of randomized patients would complete the trial, we planned to recruit 50 patients (25 patients per group).

Continuous variables are expressed as mean ± standard deviation. Baseline characteristics were compared between the two groups using the independent *t* test for continuous variables and the χ^2^ test for categorical variables. Differences in changes between the two groups were compared using the independent *t* test for normally distributed variables or the Mann–Whitney *U*-test for non-normally distributed variables. Parameters (e.g., CO, fasting glucose, and hsCRP) were assessed by two-way ANOVA. Correlation analysis was performed to evaluate the relationship between the change in CO and the change in serum fasting glucose levels (or serum hsCRP levels) after adjustment for age, sex, current smoker, hypertension (yes/no), hyperlipidemia (yes/no), diabetes (yes/no), coronary artery disease (yes/no), HF (yes/no), NYHA class, body mass index, systolic blood pressure, diastolic blood pressure, estimated glomerular filtration rate, β-blocker (yes/no), and angiotensin-converting enzyme inhibitor (yes/no). The analyses were conducted in an intention-to-treat manner. The significance level was set at *P* < 0.05. Missing values were imputed by carrying the last observed value forward. Statistical analyses were performed using SPSS software version 18.0 (SPSS, Chicago, IL, USA).

## RESULTS

### Patients

**Figure 1:**
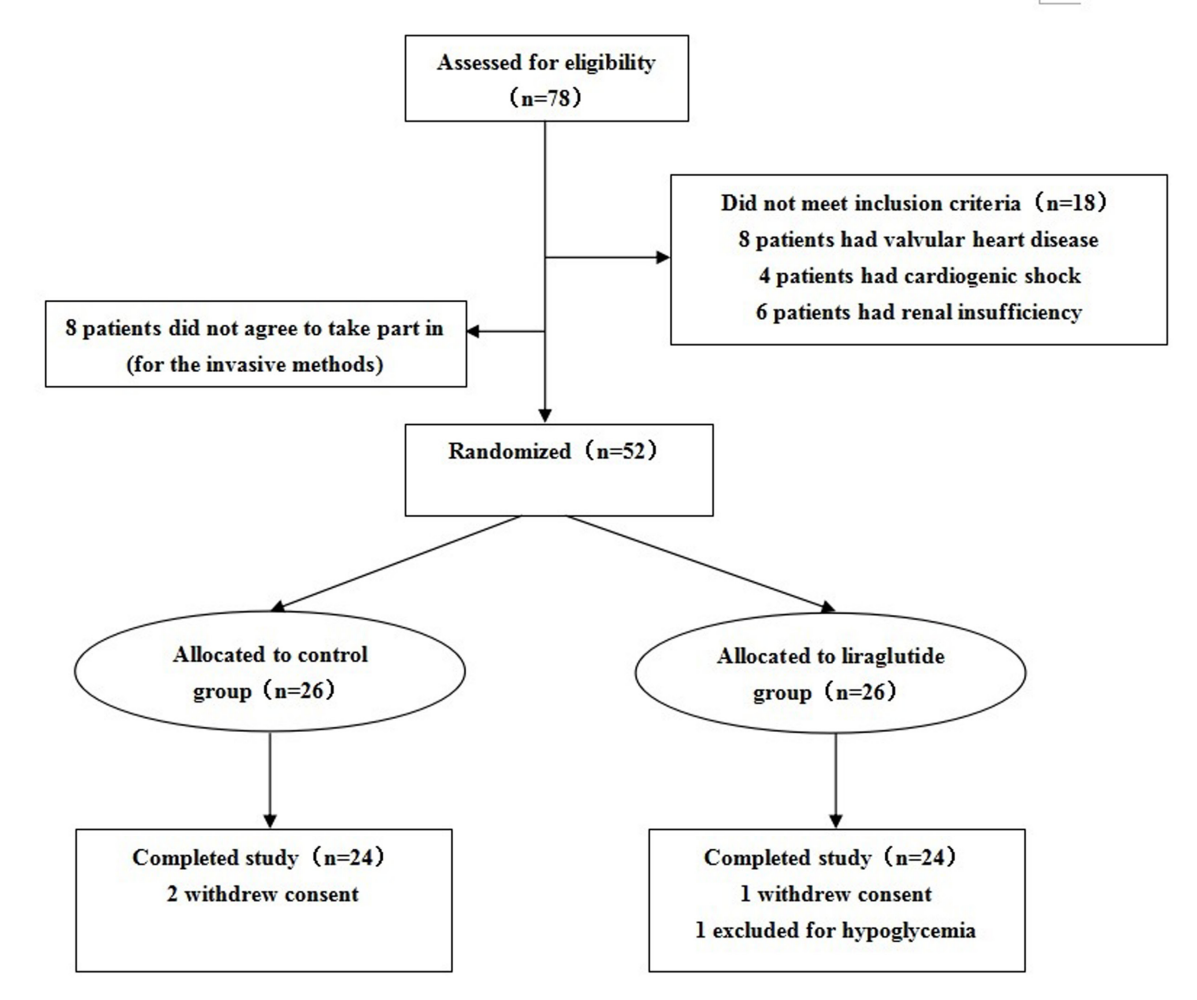
Patient flow chart

A total of 78 patients with HF were screened in this study. Of these, 52 patients were eligible and randomized 1:1 to receive either liraglutide or placebo for 7 days, and 48 patients (92%) completed the trial (Figure 1). One patient had the diagnosis of *de novo* diabetes in the liraglutide group, and two patients had *de novo* diabetes diagnosed in the control group. Two patients had diabetic retinopathy in the liraglutide group, and one patient had diabetic retinopathy in the control group. No diabetic foot complications were found in the two groups. The clinical characteristics of the two groups are shown in Tables 1 and 2. There was no significant difference in age, sex, past medical history, hemoglobin levels, or creatinine levels between the two groups (Table 1). Furthermore, the use of β-blockers, aldosterone antagonists, and angiotensin-converting enzyme inhibitors was not significantly different between the two groups.

**Table 1:** Baseline characteristics of the subjects in two treatment groups

Characteristics	Control group (*n =* 26)	Liraglutide group (*n =* 26)	*p* value
Age (years)	58.7 ± 11.4	59.1 ± 11.8	0.90
Male, *n* (%)	19 (73%)	20 (77%)	0.75
Current smoker, *n* (%)	17 (65%)	15 (58%)	0.57
Past medical history			
Hypertension, *n* (%)	16 (62%)	17 (65%)	0.77
Hyperlipidemia, *n* (%)	3 (11%)	4 (15%)	1.00
Diabetes mellitus, *n* (%)	7 (27%)	5 (19%)	0.51
Coronary artery disease, *n* (%)	20 (77%)	21 (81%)	0.73
Atrial fibrillation, *n* (%)	5 (19%)	6 (23%)	0.73
Heart failure, *n* (%)	6 (23%)	7 (27%)	0.75
NYHA classes, *n* (%)			
I	3 (12%)	2 (8%)	1.00
II	6 (23%)	7 (27%)	0.75
III	10 (38%)	9 (35%)	0.77
IV	7 (27%)	8 (30%)	0.76
BMI (kg/m^2^)	24.8 ± 3.8	25.3 ± 3.4	0.62
Systolic blood pressure, (mmHg)	115 ± 13	118 ± 14	0.43
Diastolic blood pressure, (mmHg)	64 ± 15	65 ± 13	0.79
Hemoglobin (g/dl)	13.3 ± 1.4	13.1 ± 1.3	0.59
HbA1c (%)	5.3 ± 0.4	5.4 ± 0.6	0.48
Creatinine (mg/dl)	1.0 ± 0.1	1.0 ± 0.2	0.99
eGFR (ml/min/1.73 m^2^)	83.5 ± 20.3	81.4 ± 19.5	0.71
Medications, *n* (%)			
ACEI	15 (58%)	16 (62%)	0.78
β-blocker	20 (77%)	21 (81%)	0.73
Furosemide	6 (23%)	8 (31%)	0.53
Aldosterone antagonist	11 (42%)	12 (46%)	0.78
Digoxin	4 (15%)	5 (19%)	0.71

Data are presented as mean value ± standard deviation or number or percentage (%) of patients. ACEI, angiotensin converting enzyme inhibitor; BMI, body mass index; eGFR, estimated glomerular filtration rate; HbA1c, glycosylated hemoglobin.

**Table 2:** Angiographical and procedural characteristics

Characteristics	Control group (*n =* 20)	Liraglutide group (*n =* 21)	*p* value
Number of disease vessels, *n* (%)			
Single vessel disease	4 (20%)	3 (15%)	0.94
Double vessel disease	10 (50%)	10 (47%)	0.88
Triple vessel disease	6 (30%)	8 (38%)	0.58
Culprit lesion, *n* (%)			
LAD	10 (50%)	12 (57%)	0.65
RCA	5 (25%)	4 (19%)	0.93
LCX	5 (25%)	5 (24%)	0.78
PCI	17 (85%)	19 (90%)	0.95
No. of stent per patients	1.7 ± 1.3	1.5 ± 1.4	0.64
CABG, *n* (%)	1 (5%)	1 (5%)	0.77

Data are presented as mean value ± standard deviation or number or percentage (%) of patients. LAD, left anterior descending artery; RCA, right coronary artery; LCX, left circumflex artery; PCI, percutaneous coronary intervention; CABG, coronary artery bypass grafting.

Hypoglycemia was reported in 1/26 (4%) patients in the control group and in 2/26 (8%) patients in the liraglutide group. Nausea occurred in 1/26 (4%) patients in the control group and in 3/26 (11%) patients in the liraglutide group.

### Hemodynamic variables

**Figure 2:**
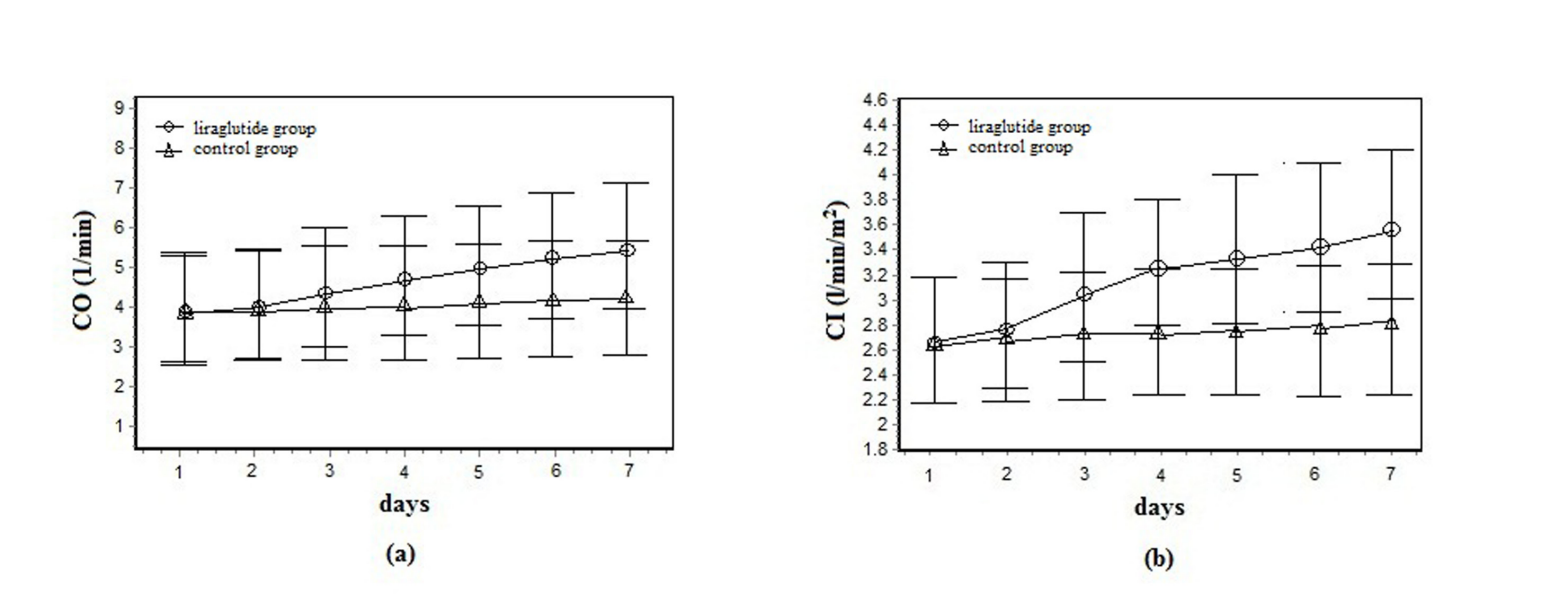
**(A)** Cardiac output (CO) in liraglutide group (*n* = 26) and control group (*n* = 26) from baseline to day 7. **(B)** Cardiac index (CI) in liraglutide group (*n* = 26) and control group (*n* = 26) from baseline to day 7.

At 7 days, the difference in change of CO (primary endpoint) between the liraglutide group and control group was +1.1 1/min (95% CI +0.1 to +2.2; *P* < 0.001) (Table 3 and Figure 2). In patients with a left ventricular ejection fraction (LVEF) < 30% at baseline (liraglutide group: *n =* 5; control group: *n =* 6), the difference in change of CO between the two groups after 7 days of treatment was +1.5 l/min (95% CI +0.2 to +2.9) (*P* < 0.001). The mean rise in the CI was significantly greater in the liraglutide group than in the control group (*P* < 0.001). Stroke volume was significantly higher in the liraglutide group compared with the control group (difference: +14.6 ml; *P* < 0.001). The difference in increase of dPmx after 7 days of treatment was +210.7 mmHg/s (liraglutide versus control groups, 95% CI −92.1 to +501.5; *P* < 0.001). However, the differences in changes in MAP and the GEDVI between the two groups were not significant.

**Table 3:** Comparison of hemodynamic variables and echocardiographic parameters between control group and liraglutide group

Parameters	Control group (*n* = 26)		Change in control group	Liraglutide group (*n* = 26)		Change in liraglutide group	*p* value
	Before treatment	7 days later		Before treatment	7 days later		
Hemodynamic variables							
HR (beats/min)	81.3 ± 8.3	75.3 ± 7.2	–5.4 (–13.1 to +6.1)	80.4 ± 8.5	74.4 ± 7.5	–6.1 (–15.9 to +5.0)	0.66
MAP (mmHg)	84.4 ± 14.7	80.3 ± 15.6	–4.1 (–19.2 to +10.9)	85.6 ± 14.9	80.5 ± 15.7	–4.9 (–19.6 to +10.1)	0.96
CO (1/min)	4.1 ± 1.4	4.4 ± 1.3	+0.3 (–0.1 to +0.6)	4.2 ± 1.5	5.3 ± 1.4	+1.4 (+0.2 to +2.8)*	< 0.001
CI (l/min/m^2^)	2.6 ± 0.7	2.9 ± 0.8	+0.3 (–0.1 to +0.5)	2.6 ± 0.6	3.4 ± 0.8	+0.8 (+0.3 to +1.3)*	< 0.001
SV (ml)	49.5 ± 11.5	54.4 ± 11.7	+5.0 (+2.0 to +8.5)	48.8 ± 10.3	67.7 ± 11.8	+19.3 (+12.2 to +26.3)*	< 0.001
GEDVI (ml/m^2^)	825.9 ± 155.8	765.3 ± 111.5	–60.5 (–361.7 to +249.5	817.8 ± 169.0	704.2 ± 109.4	–113.6 (–412.2 to +194.2)	0.53
dPmx (mmHg/sec)	701.2 ± 160.9	859.1 ± 210.5	+160.1 (–86.8 to +378.3)	708.0 ± 170.1	1080.6 ± 218.1	+380.6 (+170.6 to +585.2)*	< 0.001
CVP (mmHg)	12.8 ± 6.8	9.9 ± 4.6	–2.9 (–12.8 to +7.0)	12.6 ± 6.7	8.7 ± 3.1	–3.8 (–13.7 to +7.9)	0.74
Echocardiographic parameters							
LVEDV (ml)	115.7 ± 19.7	124.7 ± 21.5	+8.9 (+3.7 to +13.8)	117.9 ± 21.6	123.3 ± 18.7	+7.2 (+2.2 to +12.6)	0.23
LVESV (ml)	65.6 ± 8.5	69.1 ± 8.8	+4.0 (+1.3 to +6.5)	66.7 ± 7.9	57.7 ± 7.8	–10.1 (–15.2 to –5.8)*	< 0.001
LVEF (%)	42.1 ± 7.3	45.6 ± 7.6	+3.4 (+0.2 to +5.7)	42.2 ± 7.1	51.2 ± 8.1	+8.7 (+3.2 to +13.7)*	< 0.001

Values represent the mean ± standard deviation. *P < 0.05 liraglutide group vs. control group by 2-way ANOVA. MAP, mean arterial pressure; HR, heart rate; CO, cardiac output; CI, cardiac index; SV, stroke volume; GEDVI, global end diastolic volume index; dPmx, left ventricular contractile index; CVP, central venous pressure. LVEF, left ventricular ejection fraction; LVEDV, left ventricular end-diastolic volume; LVESV, left ventricular end-systolic volume.

LVEF measured by echocardiography significantly increased from 42.2% ± 7.1% to 51.2% ± 8.1% in the liraglutide group, and from 42.1% ± 7.3% to 45.6% ± 7.6% in the control group at day 7 (*P* < 0.001).

### Fasting glucose, hsCRP, interleukin-6, SOD, malondialdehyde, NO, and NOS levels

**Figure 3:**
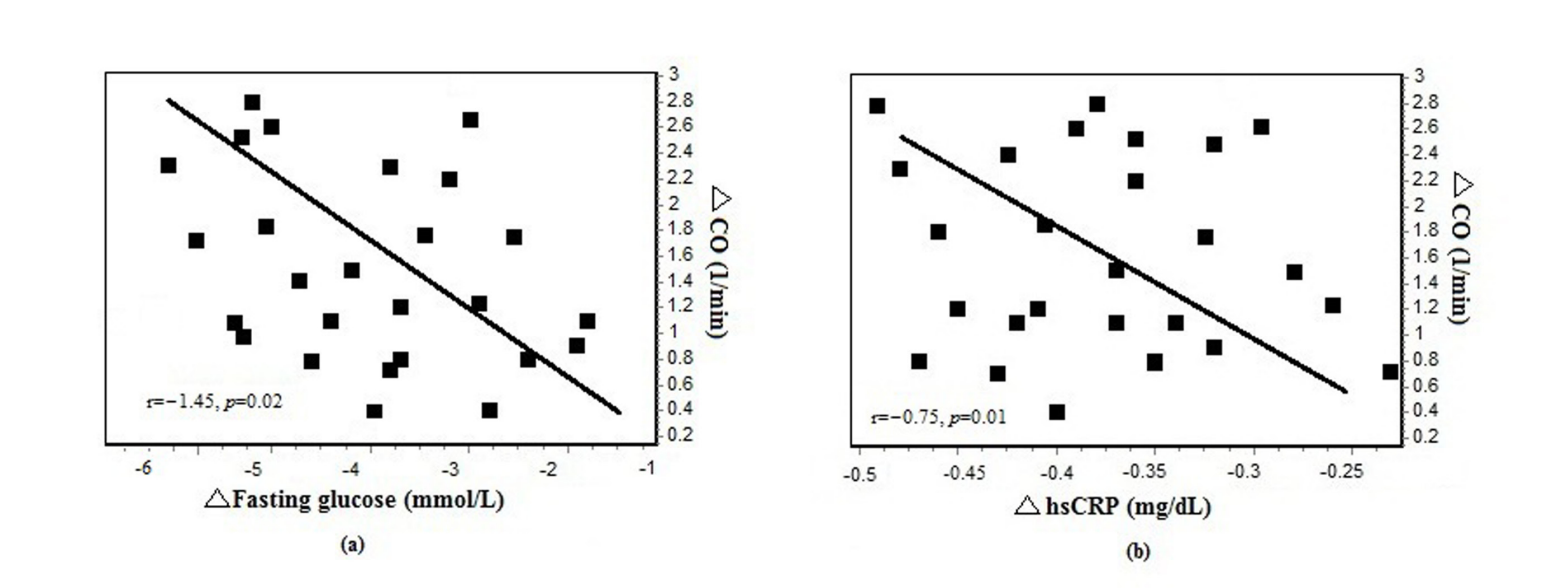
**(A)** Changes in cardiac output (CO) correlated with changes in fasting glucose in liraglutide group at 7 days (*n =* 26). (**B**) Changes in cardiac output (CO) correlated with changes in high-sensitivity C-reactive protein in liraglutide group at 7 days (*n =* 26).

Intention-to-treat analysis showed that fasting glucose levels decreased by 3.5 ± 1.6 mmol/L in the liraglutide group and by 2.3 ± 1.2 mmol/L in the control group from admission to the end of treatment (*P* = 0.01). The mean reduction in serum hsCRP levels was significantly greater in the liraglutide group than in the control group (Table 4). The difference in decrease in serum hsCRP levels between the liraglutide and control groups was −0.16 mg/dL (95% CI −0.20 to −0.11; *P* < 0.001). After 7 days of treatment, plasma SOD levels were higher and serum malondialdehyde levels were lower in the liraglutide group than in the control group. The difference in increase in serum SOD levels after 7 days of treatment was +14.2 U/mL (95% CI +4.4 to +26.2; *P* < 0.001). The change in CO was negatively correlated with the change in serum fasting glucose levels (*r =* −1.45, *P* = 0.02) and hsCRP levels (*r =* −0.75, *P* = 0.01) in adjusted analyses (Figure 3). There was also a correlation between the change in CO and the change in serum SOD levels (*r =* 0.18, *P* = 0.03).

**Table 4:** Lab investigations of patients in two treatment groups

Parameters	Control group (*n =* 26)		Change in control group	Liraglutide group (*n =* 26)		Change in liraglutide group	*p* value
	Before treatment	7 days later		Before treatment	7 days later		
Fasting blood glucose (mmol/L)	8.5 ± 3.2	6.4 ± 1.5	–2.3 (–3.5 to –1.5)	8.6 ± 3.8	5.2 ± 1.8	–3.5 (–5.5 to –1.8)*	0.01
Total cholesterol (mg/dL)	169.8 ± 25.6	151.8 ± 22.6	18.9 (–28.4 to –9.5)	175.3 ± 34.5	152.7 ± 23.8	–22.8 (–32.7 to –9.4)	0.17
Triglyceride (mg/dL)	92.2 ± 10.6	84.5 ± 10.8	–8.4 (–13.6 to –3.5)	92.8 ± 12.8	82.8 ± 9.5	–9.5 (–15.6 to –5.5)	0.43
LDL cholesterol (mg/dL)	94.8 ± 22.7	82.7 ± 17.8	–12.2 (–21.4 to –3.5)	95.1 ± 22.8	77.3 ± 18.4	–17.8 (–27.2 to –7.5)	0.29
NT–pro-BNP (pg/ml)	871.5 ± 338.6	216.8 ± 96.1	–645.9 (–871.4 to –412.6)	846.1 ± 384.6	95.5 ± 36.8	–751.6 (–1061.2 to –465.5)	< 0.001
hsCRP (mg/dL)	1.08 ± 0.48	0.92 ± 0.38	–0.18 (–0.25 to –0.08)	1.06 ± 0.45	0.72 ± 0.35	–0.35 (–0.48 to –0.19)*	< 0.001
Interleukin-6 (pg/mL)	14.7 ± 3.2	8.4 ± 2.5	–6.8 (–9.5 to –3.4)	15.2 ± 3.8	7.4 ± 2.5	–7.9 (–11.4 to –3.2)	0.27
SOD (U/mL)	105.7 ± 22.8	112.7 ± 21.5	+6.5 (+2.8 to +9.4)	102.8 ± 21.4	124.5 ± 20.6	+20.7 (+9.5 to +32.8)*	< 0.001
Malondialdehyde (nmol/mL)	6.08 ± 1.84	6.02 ± 1.55	–0.06 (–0.09 to –0.02)	6.07 ± 1.93	5.92 ± 1.77	–0.15 (–0.23 to –0.07)*	< 0.001
NO (umol/L)	51.7 ± 8.4	59.4 ± 9.3	+8.3 (+3.5 to +12.8)	52.1 ± 8.5	61.2 ± 9.2	+9.5 (+5.2 to +13.4)	0.28
NOS (U/mL)	8.84 ± 0.14	9.85 ± 0.13	+0.98 (+0.52 to +1.38)	8.95 ± 0.12	10.2 ± 0.18	+1.11 (+0.52 to +1.63)	0.31

Values represent the mean ± standard deviation. *P < 0.05 liraglutide group vs. control group by 2-way ANOVA. LDL, low- density lipoprotein; hsCRP, high-sensitivity C-reactive protein; SOD, superoxide dismutase; NO, nitric oxide; NOS, nitric oxide synthase; NT-pro-BNP, N-terminal pro-brain natriuretic peptide.

When age, smoking, hypertension (yes/no), hyperlipidemia (yes/no), diabetes (yes/no), coronary artery disease (yes/no), HF (yes/no), NYHA class, body mass index, systolic blood pressure, diastolic blood pressure, and use of liraglutide were considered as explanatory variables, and improvement in CO was set as the dependent variable, administration of liraglutide was consistently identified as a significant determinant for improvement in CO using multivariate regression analysis (*P* = 0.01) (Table 5).

**Table 5:** Independent variables for the improvement in cardiac output

Independent variables	Regression coefficient	hazard ratio (95% CI)	*p* value
Age	–0.022	0.95 (0.92–1.01)	0.28
Smoking	–0.215	0.91 (0.82–1.21)	0.37
Systolic blood pressure	–0.021	0.96 (0.86–1.11)	0.51
Diastolic blood pressure	–0.016	0.82 (0.73–1.04)	0.57
Administration of liraglutide	2.177	1.64 (1.10–2.26)	0.01
HsCRP	–0.622	0.68 (0.37–0.81)	0.00
Malondialdehyde	–0.019	0.95 (0.92–0.98)	0.03

hsCRP, high-sensitivity C-reactive protein.

### Events during the 3-month follow-up

During 3 months of follow-up, the incidence of MACE was not significantly lower in the liraglutide group than in the control group (*P* > 0.05). There were no significant differences in the incidence of myocardial infarction, hospitalization for HF, and cardiac death between the groups (Table 6).

**Table 6:** Clinical outcome 3 months after initial treatment

	Control group (*n =* 26)	Liraglutide group (*n =* 26)	*p* value
MACE at 3 months, *n* (%)	5 (19%)	3 (11%)	0.70
Myocardial infarction, *n* (%)	2 (7%)	1 (4%)	1.00
Hospitalization for heart failure, *n* (%)	2 (7%)	1 (4%)	1.00
Cardiac death, *n* (%)	1 (4%)	0	0.50

MACE, major adverse cardiovascular event.

## DISCUSSION

We reported the effects of liraglutide on hemodynamic parameters in patients with HF. A significant difference in CO was observed between the two treatment groups. Additionally, liraglutide caused favorable changes in markers of inflammation and oxidative stress.

There are several important differences between our study and other studies [3, 5, 6, 16]. First, GLP-1 agonists have been extensively studied for improvement of cardiovascular function in preclinical and clinical models. The current study focused on hemodynamic parameters (CO, dPmx, GEDVI) in patients with HF at 7 days, while our previous study focused on LVEF in patients with acute myocardial infarction at 3 months [14, 15]. Second, a previous study by Nikolaidis *et al.* was short term and non-randomized [3]. The first randomized study of GLP-1 in HF by Halbirk *et al.* could not demonstrate an improvement in cardiac function [16]. Third, in our previous study, a short 7-day course of liraglutide in patients with non-ST-segment elevation myocardial infarction was associated with improvement in LVEF [14]. A 7-day treatment period was also chosen in the current study. Fourth, Nikolaidis *et al.* observed significant beneficial effects of GLP-1 on global LVEF in patients with and without diabetes [3]. We decided to choose liraglutide as the first choice of treatment in HF patients with and without diabetes. Fifth, the effect of liraglutide on LVEF was substantial in our study. Neither LIVE nor FIGHT showed a positive effect of liraglutide on LVEF [5, 6]. This difference in finding among studies might be related to the study population; patients with acute myocardial infarction were included in this study. Sixth, β-blockers, angiotensin-converting inhibitors, angiotensin-II receptor agonists, and diuretics are the cornerstone in HF treatment. Blood pressure was not high in patients in our study, and some patients did not receive an optimal dose of β-blockers or angiotensin-converting inhibitors. This could partly explain the discrepancy in findings between our study and recent studies of HF [5, 6]. Additionally, Kumarathurai found that liraglutide increased heart rate in patients with coronary artery disease after 12 weeks [17]. Long-term treatment of patients with type 2 diabetes with GLP-1 showed improvement in glycemic control coupled with weight loss and lower blood pressure [18]. In our study, no effects of liraglutide on blood pressure and heart rate were observed in patients. These results may be partially explained by our short-term treatment with liraglutide.

The effects of GLP-1 on the cardiovascular system have been recently studied. A study in anesthetized pigs showed that pretreatment with GLP-1 decreased accumulation of lactate and pyruvate in ischemic myocardium, but had no effect on functional parameters [19]. In patients with type 2 diabetes and recent acute coronary syndrome, the addition of lixisenatide to usual care did not significantly alter the rate of major cardiovascular events or other serious adverse events [20]. However, Noyan-Ashraf *et al.* found that liraglutide significantly improved CO (12.4 ± 0.6 versus 9.7 ± 0.6 ml/min; *P* = 0.002) in mice [21]. In our previous study, the difference in change in LVEF between the liraglutide group and the control group was + 4.7% (95% CI + 0.7% to + 9.2% *P* = 0.009) [14]. In the current study, CO was significantly higher in the liraglutide group compared with control group. The difference in change of LVEF between the liraglutide group and control group was significant (+5.7%) in patients with HF. To the best of our knowledge, this is the first randomized trial to assess the effect of liraglutide in treatment of patients with HF using the PiCCO system.

Femoral artery dPmx provides reliable estimations of left ventricular systolic function [22]. Changes in dPmx accurately reflect changes in left ventricular contractile function in patients [23]. GLP-1 markedly improves post-ischemic cardiac contractile function in rats [24]. Poornima *et al.* found that chronic GLP-1 infusion improved left ventricular systolic function [25]. In our study, dPmx was also significantly increased in the liraglutide group. The transpulmonary thermodilution GEDVI behaves as an indicator of cardiac preload [26]. Some studies have suggested that GLP-1 stimulates solute-free water excretion by the human kidney and decreases fluid overload [27, 28]. Although the difference in change in the GEDVI between the liraglutide and control groups was not significant, the decrease observed in the liraglutide group was greater than that in the control group (−113.6 ± 301.2 ml/m^2^ versus −60.5 ± 313.5 ml/m^2^).

### Potential mechanisms

Plasma glucose, blood pressure, and lipids are continuous variables that exert a dose-dependent effect on cardiovascular risk [29]. GLP-1 is an incretin hormone, which promotes myocardial glucose uptake [30]. GLP-1 might decrease glucose levels, improve cardiovascular risk, and enhance recovery of cardiac function [31]. HF is associated with inflammation and oxidative stress [32]. GLP-1 has been reported to ameliorate inflammation [33, 34]. Liraglutide might reduce inflammation and increase left ventricular contractile function and CO. GLP-1 can reduce oxidative stress [34]. Our results suggest that liraglutide might improve left ventricular function by reducing oxidative stress. This mechanism needs to be investigated in further studies.

### Study limitations

This study has several limitations. First, the main limitation of this study is that it was from a single center and it had a small study size. Second, hemodynamic measurements were performed invasively with central venous and arterial catheters, and the results might be difficult to translate in routine clinical practice. Third, patients with moderate signs of HF were selected (i.e., ejection fraction < 50%). Therefore, data may be translated with difficulty to more severe conditions. Fourth, the genetic background should be considered. Some important polymorphisms are associated with endothelial dysfunction and coronary artery disease, including calcium/calmodulin-dependent kinase IV [35], glycoprotein IIIa PIA2 [36], and G-protein-coupled receptor kinases [37]. Fifth, liraglutide usually increases insulin secretion in hyperglycemia. Therefore, postprandial blood glucose and serum insulin levels should have been measured in our study.

## CONCLUSIONS

GLP-1 may be associated with improvement in left ventricular function in patients with HF. This finding needs to be confirmed by larger invasive trials.
